# Impact of Restricted Contact between Grandparents and Grandchildren during the COVID-19 Pandemic

**DOI:** 10.1093/geroni/igab046.3615

**Published:** 2021-12-17

**Authors:** Verena Klusmann, Sara Cengiz, Carina Materna, Christian Spreckels

**Affiliations:** 1 University of Konstanz, Berlin, Berlin, Germany; 2 University of Hamburg, University of Hamburg, Hamburg, Germany

## Abstract

To combat the COVID-19 pandemic strict contact restrictions have been imposed on institutions for both older and younger people, social structures have been locked down, families have been urged to reduce contact with older relatives, and people over 65 have been temporarily banned from their workplaces and from attending events, both in public and private spaces. These measures are assumed to have a number of psychosocial consequences. For this questionnaire study, 268 pupils (7-to-10 years-old) of nine different schools in Hamburg, Germany, with different social index were asked about how they experienced, perceived, and behaved during the COVID-19 pandemic. 75% of the children reported on restricted contact to their grandparents: While 41% did not meet their grandparents at least for a certain time at the beginning of the pandemic, 34% did not meet their grandparents during the whole first year of the pandemic. Of those who met their grandparents, 25% kept physical distance to them. These contact restrictions were significantly higher in schools with a lower social index, chi2(8)=15.49, p=.05. Those children who never met their grandparents also reported on higher perceived stress, t(220)=-2.37, p=.019, d=-.33, tended to have lower subjective well-being, t(223)=-1.73, p=.09, d=-.24, and had higher risk perceptions concerning COVID-19 infections, t(223)=-2.18, p=.03, d=-.31. Hence social isolation and loneliness is not only an issue for older people themselves, but contact restrictions also potentially increase the stress load and impair the well-being of children who have to do without support and care of their grandparents in sensitive developmental phases.

